# Azithromycin and cefixime combination versus azithromycin alone for the out-patient treatment of clinically suspected or confirmed uncomplicated typhoid fever in South Asia: a randomised controlled trial protocol

**DOI:** 10.12688/wellcomeopenres.16801.1

**Published:** 2021-08-11

**Authors:** Abhishek Giri, Abhilasha Karkey, Sabina Dongol, Amit Arjyal, Archana Maharjan, Balaji Veeraraghavan, Buddhi Paudyal, Christiane Dolecek, Damodar Gajurel, Dung Nguyen Thi Phuong, Duy Pham Thanh, Farah Qamar, Gagandeep Kang, Ho Van Hien, Jacob John, Katrina Lawson, Marcel Wolbers, Md. Shabab Hossain, M Sharifuzzaman, Nantasit Luangasanatip, Nhukesh Maharjan, Piero Olliaro, Priscilla Rupali, Ronas Shakya, Sadia Shakoor, Samita Rijal, Sonia Qureshi, Stephen Baker, Subi Joshi, Tahmeed Ahmed, Thomas Darton, Tran Nguyen Bao, Yoel Lubell, Evelyne Kestelyn, Guy Thwaites, Christopher M. Parry, Buddha Basnyat

**Affiliations:** 1 Oxford University Clinical Research Unit-Nepal, Patan Academy of Health Scineces, Lalitpur, Bagmati, 44700, Nepal; 2 Centre for Tropical Medicine and Global Health, Nuffield Department of Medicine, University of Oxford, Oxford, UK; 3 Patan Academy of Health Sciences, Lalitpur, Bagmati, 44700, Nepal; 4 Christian Medical College, Vellore, Tamil Nadu, 632004, India; 5 CIvil Service Hospital, Kathmandu, 44600, Nepal; 6 Oxford University Clinical Research Unit, Ho Chi Minh City, Vietnam; 7 Aga Khan University Hospital, Karachi, 74800, Pakistan; 8 International Centre for Diarrhoeal Disease Research (icddr, b), Dhaka, Bangladesh; 9 Mahidol Oxford Tropical Medicine Research Unit (MORU), Bangkok, 10400, Thailand; 10 Department of Medicine, University of Cambridge, Cambridge, UK; 11 Department of Infection, Immunity and Cardiovascular Disease, University of Sheffield, South Yorkshire, UK; 12 Department of Clinical Sciences, Liverpool School of Tropical Medicine, Liverpool, UK

**Keywords:** Enteric fever, South Asia, RCT, cefixime, azithromycin

## Abstract

**Background: **Typhoid and paratyphoid fever (enteric fever) is a common cause of non-specific febrile infection in adults and children presenting to health care facilities in low resource settings such as the South Asia.  A 7-day course of a single oral antimicrobial such as ciprofloxacin, cefixime or azithromycin is commonly used for its treatment. Increasing antimicrobial resistance threatens the effectiveness of these treatment choices. We hypothesize that combined treatment with azithromycin (active mainly intracellularly) and cefixime (active mainly extracellularly) will be a better option for the treatment of typhoid fever in South Asia.

**Methods:** This is a phase IV, international multi-centre, multi-country, comparative participant-and observer-blind, 1:1 randomised clinical trial. Patients with suspected uncomplicated typhoid fever will be randomised to one of the two interventions: Arm A: azithromycin 20mg/kg/day oral dose once daily (maximum 1gm/day) and cefixime 20mg/kg/day oral dose in two divided doses (maximum 400mg bd) for 7 days, Arm B: azithromycin 20mg/kg/day oral dose once daily (max 1gm/day) for 7 days AND cefixime-matched placebo for 7 days. We will recruit 1500 patients across sites in Bangladesh, India, Nepal and Pakistan. We will assess whether treatment outcomes are better with the combination after one week of treatment and at one- and three-months follow-up.

**Discussion:** Combined treatment may limit the emergence of resistance if one of the components is active against resistant sub-populations not covered by the other antimicrobial’s activity. If the combined treatment is better than the single antimicrobial treatment, this will be an important result for patients across South Asia and other typhoid endemic areas.

**Clinicaltrials.gov registration:** NCT04349826 (16/04/2020)

## Introduction

Typhoid (caused by *Salmonella enterica* serovar Typhi) and paratyphoid fever (caused by *Salmonella enterica* serovar Paratyphi A), collectively referred to as typhoid fever in this protocol, are common causes of non-specific febrile infection in adults and children presenting to health care facilities in low resource settings such as the South Asia region ^1– 4^. South Asia has been described as the largest hub for typhoid fever in the world and antimicrobial resistance has become a critical issue ^5^. In the last 20 years, treatment of typhoid fever with a 7-day course of a single oral antimicrobial, such as ciprofloxacin, cefixime or azithromycin, given in an out-patient setting has led to patient recovery in 4 to 6 days without the need for expensive hospitalization. Increasing antimicrobial resistance in Asia and sub-Saharan Africa threatens the effectiveness of these treatments and increases the risk of prolonged illness and severe disease.

The fluoroquinolone class of antimicrobials have been a common choice to treat typhoid fever across South Asia during the last two decades. Now low and high-level resistance is so widespread that the entire class is no longer a reliable treatment choice ^2, 6^. Cefixime and azithromycin are commonly recommended alternatives and widely used ^7– 11^, but the conflicting results from small randomised controlled trials (RCTs) with cefixime and azithromycin in typhoid fever leave many clinicians unsure about the optimum antimicrobial treatment for typhoid fever in this region.

The outcome for patients treated with cefixime (10–20mg/kg/day for 7–14 days) in typhoid fever are particularly mixed with prolonged fever clearance times of 6–8 days, clinical failure rates of between 6% and 27% and microbiological failure rate between 0% and 4.5% ^12– 15^. The reported fever clearance times with azithromycin treatment (10–20 mg/kg/day for 5–7 days) is usually 4–6 days and clinical failure rates range from 0% to 18% with a microbiological failure rate from 0% to 3.2% ^7, 16– 23^. A number of the studies with azithromycin have demonstrated a delayed microbiological clearance, indicated by positive blood culture during the treatment course ^17, 19– 21, 23^. Azithromycin activity is predominantly intracellular with studies from Vietnam indicating that up to one third of *S.* Typhi may be found in the extracellular compartment ^24, 25^. The low levels of azithromycin in plasma may be insufficient to ensure adequate killing activity ^26^. In contrast, the extended-spectrum cephalosporin cefixime is predominantly active in the extracellular compartment although *in-vitro* evidence also suggests some intracellular activity ^27^. The relative lack of intracellular activity by cefixime may be one reason for the variable treatment results and prolonged fever clearance times in patients with typhoid infection. For each of these antimicrobials, the exposure of the extracellular or intracellular bacteria to sub-therapeutic drug levels will increase its likelihood of resistance emergence.

### Rationale for the Azithromycin Cefixime Trial (ACT): an RCT in South Asia

We hypothesize that a combined treatment with azithromycin, active mainly intracellularly, and cefixime, active mainly extracellularly, will be a better option for the treatment of typhoid fever in South Asia. There is some clinical evidence for this. The clinical response to treatment in 37 Israeli travellers returning from Nepal with paratyphoid fever was significantly better when azithromycin was combined with ceftriaxone in comparison to ceftriaxone alone with the fever clearance times reduced from six to three days ^28^. In an RCT of 105 adults with confirmed typhoid fever in Nepal, a combination of azithromycin and cefixime for out-patients and azithromycin and ceftriaxone for in-patients was superior to azithromycin alone with shorter fever clearance times ^17^. Unpublished data suggests that there is no evidence for significant antagonism against *S*. Typhi *in-vitro* with the combination of azithromycin and ceftriaxone/cefixime (Veeraraghavan, unpublished data, Table 1).

**Table 1.** *In-vitro* antimicrobial interactions between azithromycin and cefixime for recent clinical blood culture isolates of *S*. Typhi.

Antimicrobial	Checkerboard assay, n (%) Total 100 isolates			Time kill assay, n (%) Total; 50 isolates		
	Synergy	Indifference	Antagonism	Synergy	Indifference	Antagonism
Azithromycin – ceftriaxone	14 (14)	85 (85)	0	12 (24)	34 (68)	4 (8)
Azithromycin – cefixime	6 (6)	94 (94)	0	8 (16)	38 (76)	4 (8)

### Study design

This is a phase IV, international multi-centre, multi-country, comparative participant and observer-blind, 1:1 randomised clinical treatment trial. Patients with suspected uncomplicated typhoid fever will be randomised to one of the two interventions: Arm A: azithromycin 20mg/kg/day oral dose once daily (maximum 1gm/day) AND cefixime 20mg/kg/day oral dose in two divided doses (maximum 400mg bd) for 7 days, Arm B: azithromycin 20mg/kg/day oral dose once daily (max 1gm/day) for 7 days AND cefixime-matched placebo for 7 days. The drug dosing table can be found as extended data ^29^. The ACT-South Asia study aims to compare a combination of azithromycin and cefixime with azithromycin alone in the outpatient treatment of clinically suspected and confirmed uncomplicated typhoid fever. We will recruit 1500 patients across sites in Bangladesh, India, Nepal and Pakistan. A placebo (sugar pill) will be used instead of cefixime in the single drug arm so that neither the patient nor the study team know which patient is receiving which treatment. We will assess whether treatment outcomes are better with the combined treatment after one week and at one and three month follow-up. 

### Study sites

1. Oxford University Clinical Research Unit (OUCRU)- Patan Academy of Health Sciences, Kathmandu, Nepal. Recruitment will take place in the out-patient department of Patan Hospital, the Civil Services Hospital, and Kathmandu, Nepal-Korea Friendship Hospital, Bhaktapur.
2. Aga Khan University (AKU) Hospital. In addition to the main AKU campus, recruitment will take place at the National Institute of Child Health (NICH), two AKU secondary care Hospitals and the Civil Hospital, Karachi.
3. The Christian Medical College, Vellore, South India. Recruitment will take place from the Paediatrics Department, CMC, Anatapura Health Centre, and Chinnalapuram Community Health Centre, CMC.
4. The International Centre for Diarrheal Disease Research, Bangladesh (iccdr,b). Recruitment will take place at the out-patient department in the hospital.

## Protocol

**Figure 1.**
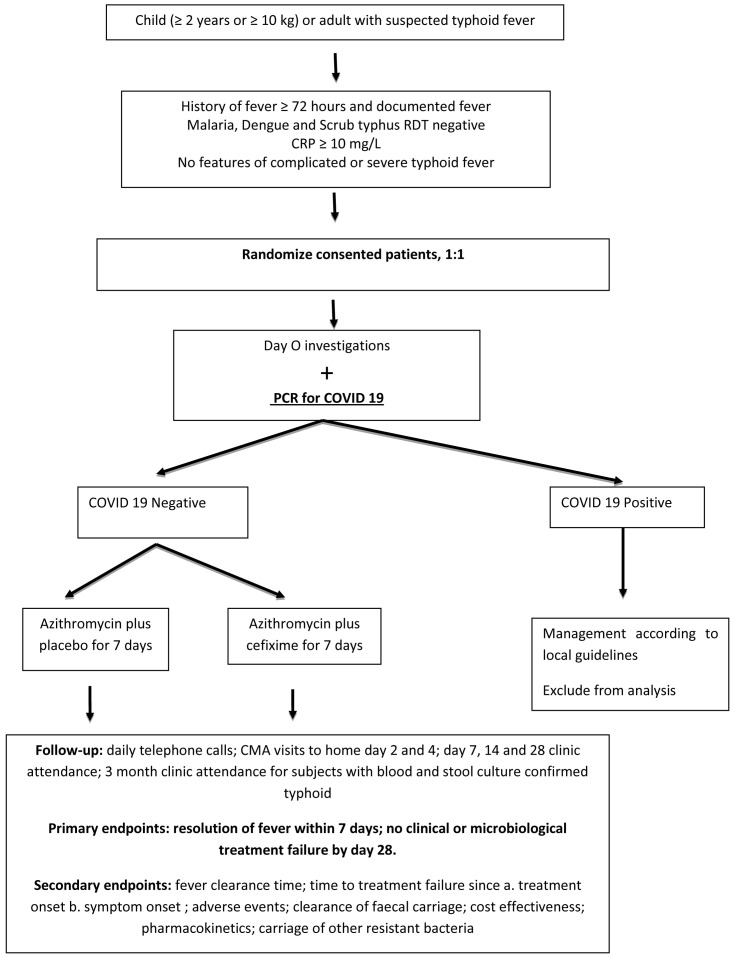
Trial schema. RDT= Rapid diagnostic test; CRP=C-reactive protein; PCR=polymerase chain reaction; COVID-19=coronavirus disease 2019; CMA=community medical auxiliaries

This trial was registered on clinicaltrials.gov on the 16 ^th^ April 2020 (NCT04349826). This protocol has been written according to the SPIRIT guidelines ^29^. Figure 1 shows the study flowchart.

### Objectives

***Primary objective***

To determine whether combined treatment with seven days of an azithromycin and cefixime is superior to seven days of azithromycin and placebo in preventing treatment failure in patients with uncomplicated typhoid fever.

***Secondary objectives***

- To compare the fever clearance time (FCT) in patients in each treatment arm
- To compare the time from onset of treatment to treatment failure in patients in each treatment arm
- To compare the time from onset of symptoms to treatment failure in patients in each treatment arm
- To compare the time from onset of symptoms to treatment failure in patients in each treatment arm
- To compare the occurrence of adverse events in each treatment arm
- To compare the clearance of faecal carriage of *S*. Typhi or *S.* Paratyphi in the patients with blood culture confirmed typhoid fever at onset in each treatment arm
- To compare the cost-effectiveness of the treatment in each treatment arm

***Exploratory objectives***

- To determine the pharmacokinetics of each drug in the two treatment arms
- To determine the diagnosis in participants who do not have blood culture confirmed typhoid fever
- To compare the faecal micro biome of patients with confirmed and suspected typhoid fever

***Endpoints***

*Primary endpoint*

A composite outcome of treatment failure by the 28th day after the initiation of treatment will be defined by either of the following events: 1.Clinical failure: persistence of fever on day 7 (168 h) post treatment initiation OR The need for rescue treatment as judged by the Trial Clinician OR The development of any complication (e.g., clinically significant bleeding, fall in the Glasgow Coma Scale score, perforation of the gastrointestinal tract) OR Syndromic enteric fever relapse within 28 days of initiation of treatment. 2.Microbiological failure: a positive blood-culture for *S.* Typhi or *S*. Paratyphi on day 7 of treatment regardless of the presence of fever (microbiological failure) OR blood culture-confirmed typhoid fever relapse within 28 days of initiation of treatment.

*Secondary endpoints*

1. The fever clearance time (FCT) will be the time from the first dose of a study drug until a temperature of < 37.5°C (axillary); < 38.0°C (oral) has been achieved for at least 48 hours

2. The time to treatment failure will be the time from the first dose of a study drug until an event occurs defined as a treatment failure

3. The time to treatment failure will be the time from the day of the first symptom until an event occurs defined as a treatment failure. As subjects are only under observation from the initiation of treatment onwards, they will be considered as left-truncated at that timepoint.

4. Adverse events will be graded (grade 3/4 adverse events, serious adverse events, adverse events of any grade leading to modification of study drug dose or interruption/early discontinuation)

5. Positive culture of faeces sample for *S*. Typhi or *S*. Paratyphi

6. The incremental cost-effectiveness ratio (ICER) will comprise of the total costs per case, real outpatient and in-patient costs, total direct and indirect costs for the family and healthcare system and health outcomes converted to disability adjusted life years (DALYs). The cost per DALY averted will be compared against multipliers of the gross domestic product (GDP)/capita in each of the four countries to establish the cost-effectiveness of the combination regimen.

7. Additional diagnostic tests on the samples collected from participants who do not have blood culture confirmed typhoid fever

8. Analysis of the faecal microbiome in collected faecal samples

*Assessment of endpoints*

Participants, or their family members, will be trained how to monitor the temperature and then contacted on a twice daily basis by telephone for monitoring and recording of temperature and enquiry using symptom checklist. Temperature and symptom logs will be maintained during the twice daily telephone calls to measure fever clearance time and when the patient is seen on day 7. Each enrolled subject will measure temperature either in the axillary area or orally and stick to one method and not interchange so the documentation will not be confusing. If there is persistence of fever and/or symptoms on day 7 the patient will be reassessed, rescue treatment administered if appropriate and the patient will be judged to be a treatment failure. In addition, adverse events or signs of any complication (using a check list) developing at any time will be closely monitored and patients will be referred to the hospital if necessary, under the supervision of one of the trial physicians.

Patients still febrile and unwell on or before day 7 will be reassessed. In case of treatment failure a decision to admit or continue treatment as an outpatient will be made by the site Principal Investigator (PI). The choice of antibiotics used for cases of treatment failure, will depend on the initial report of blood culture if positive or the prevailing antibiotic sensitivity patterns if negative. The site PI will decide if rescue treatment is required. This may be ceftriaxone 60–80 mg/kg once daily (Max 4gm od) or meropenem 10–20 mg/kg three times daily or 0.5–1gm three times daily for body weight ≥ 50kg) according to local guidelines and follow-up will continue (in hospital if necessary).

Severe or complicated typhoid fever will be defined if any of the following develop:

•   Clinically significant gastrointestinal bleeding

•   Fall in the Glasgow Coma Scale score (delirium, obtundation or coma)

•   Perforation of the gastrointestinal tract (confirmed at surgery)

•   Haemodynamic shock (systolic blood pressure < 90mmHg and/or diastolic blood pressure < 60 mmHg associated with tissue hypoperfusion

•   Myocarditis (tachycardia or bradycardia with an associated abnormality of the electrocardiogram (ECG) or ultrasound evidence of a pericardial effusion)

•   Hepatitis (indicated by jaundice and/or hepatomegaly with abnormal levels of aspartate aminotransferase (AST) (>10 × normal range) or alanine aminotransferase (ALT) (>10 × normal range))

•   Clinical diagnosis of cholecystitis (right upper quadrant pain and tenderness without evidence of hepatitis; ultrasound appearance consistent with cholecystitis)

•   Pneumonia (respiratory symptoms and new chest X-ray infiltrates)

A positive culture of *S.* Typhi or *S.* Paratyphi from blood collected on day 7, regardless of the presence of fever, will be judged to be treatment failure. Rescue treatment will be administered if necessary at the discretion of the site PI. Similarly, blood culture positive or syndromic relapse of typhoid fever during the follow-up through until day 28 will be judged a treatment failure and further treatment will be given under the guidance of the site PI. Participants will be followed-up approximately until resolution. If the illness is not resolved at that time of follow-up, an additional follow-up will be arranged

Faecal carriage of *S.* Typhi or *S.* Paratyphi in the patients who were blood /stool culture positive for these organisms at entry to the study will be determined at 7 days, 14 days, 28 days and 3 months to check for clearance. If faecal carriage is still present at the 3 month follow up visit the Site PI will decide the need for further follow-up and treatment.

We will collect data on health facility and household costs and loss of income during the illness and up to 28 days to enable a cost of illness analysis for typhoid fever in these settings, as well as a comprehensive economic evaluation of the treatment strategies. The difference in direct purchase costs of the treatment strategies in the four countries are modest (e.g. a full course of azithromycin in the four countries is ~$5, and an additional course of cefixime would also cost ~$ 5). Therefore when considering these costs alone, any clinical benefits associated with the combined regimen may imply that the combined regimen is cost-effective. The economic benefits of the combined regimen, however, may extend further to include other health facility and household costs averted as most people in the subcontinent do not have health insurance; demonstrating these will be useful for policy guidance. We will therefore collect data on health facility and household costs and loss of income during the illness and up to 28 days to enable a cost of illness analysis for typhoid fever in these settings, as well as a comprehensive economic evaluation of the treatment strategies. The analysis will take a societal perspective and include the costs of AMR per antibiotic consumed using methods from a recent publication on this topic ^12^. We will measure the incremental cost-effectiveness ratio (ICER) which will comprise of the total costs per case, real outpatient and in-patient costs, total direct and indirect costs for the family and healthcare system and health outcomes converted to Disability Adjusted Life Years (DALYs). The cost per DALY averted will then be compared against multipliers of the GDP/capita in each of the four countries to establish the cost-effectiveness of the combination regimen.

The end of trial is the point at which the last participant has completed their follow-up period, all the data has been entered and queries resolved, the last sample has been processed and the database locked.

### Study procedures

***Inclusion criteria***

•   A history of fever at presentation for ≥ 72 hours and a documented fever (≥37.5 ^o^C (axillary) or ≥38 ^o^C (oral))

•   Age ≥ 2 years (and ≥ 10kg) to 65 years

•   No clear focus of infection on initial clinical evaluation

•   Malaria rapid diagnostic test (RDT) negative; dengue non-structural protein NS1 RDT negative; scrub typhus RDT negative; C-reactive protein (CRP) rapid test ≥10 mg/L

•   Able to take oral treatment

•   Able to attend for follow-up and can be contacted by telephone

•   Written fully informed consent to participate in the study including assent for children in addition to parental/legal guardian consent.

***Exclusion criteria***

The participant may not enter the trial if ANY of the following apply:

•   History of fever for >14 days

•   Pregnant or positive pregnancy test or breast-feeding

•   Presence of clinical symptoms or signs indicating a focal infection such as pneumonia; urinary infection, meningitis, eschar

•   Obtundation, haemodynamic shock, visible jaundice, gastrointestinal bleeding or any signs of severe disease that may require immediate hospitalisation

•   Being treated for tuberculosis (TB) or human immunodeficiency virus (HIV) or severe acute malnutrition

•   Patients with cardiac disease

•   Patient requiring intravenous antibiotics for any reason

•   Previous history of hypersensitivity to any of the treatment options

•   Either of the trial drugs are contraindicated for any reason (e.g. drug interactions)

•   Has received azithromycin or cefixime in the last five days

•   Receiving another antimicrobial and responding clinically to the treatment as judged by the attending clinician.

•   Being on another drug (for example certain kinds of anti-depressants, or anti-convulsants) that may also cause prolonged QT interval

•   Positive polymerase chain reaction (PCR) test for coronavirus disease 2019 (COVID-19) before or after randomisation

***Screening and eligibility.*** The non-specific clinical presentation of typhoid fever can make it difficult to distinguish typhoid from other causes of an acute undifferentiated febrile illness such as malaria and dengue based on clinical history, physical examination and initial laboratory investigations alone ^28, 30^. There are no satisfactory rapid diagnostic tests for typhoid fever ^31^, and patients are treated for presumed typhoid fever (in high typhoid fever incidence areas) or fever without a focus that require antimicrobials (in low enteric fever incidence areas). We will use the RDTs available for other common causes of fever in our region to rule out malaria, dengue, and scrub typhus, and a CRP cut-off to select the sub-group of patients more likely to have a bacterial infection such as typhoid fever. In a large fever study from South East Asia, a CRP at a threshold of 10mg/L had sensitivity for detecting bacterial infections of 95% with a specificity of 49% ^32^. In 251 adults and children with blood culture positive typhoid fever in Vietnam, 242 (96.4%) had a CRP ≥10mg/L at presentation (CM Parry, unpublished data). In addition, with the appearance of COVID-19, this disease will have to be kept in the differential diagnosis as a cause of suspected or confirmed typhoid fever. We plan to do a PCR test for COVID-19 after randomization of our patients as there is usually a 24 hour test turnaround time. If the patient tests positive, the patient will be taken out of the study and followed up in a regular fashion as per the local hospital rules. The screening for COVID-19 will be site specific and may be based on reverse transcription polymerase chain reaction (RT-PCR) or antigen testing as per the local guidelines of the country.

***Informed consent.*** Prospective, written informed consent will be obtained from the participant, or the parent or person with legal responsibility (including legal authorities) for a child (if participant’s age is ≤ 18 years old), after explanation of the aims, methods, benefits and potential hazards of the trial and before trial specific procedures are performed. The consent forms can be found as extended data ^29^. Any patients potentially eligible for the study will be screened by the research nurse (RN) and community medical auxiliaries (CMA). Those meeting the criteria will be approached for informed consent by the study doctor.

Written and verbal versions of the participant information and informed consent will be presented to the participant, or the parent or person with legal responsibility for a child, detailing: the exact nature of the trial; what it will involve for the participant; the implications and constraints of the protocol; the known side effects and any risks involved in taking part. It will be clearly stated that the participant, or the parent or person with legal responsibility for a child, is free to withdraw from the trial at any time for any reason without prejudice to future care, without affecting their legal rights and with no obligation to give the reason for withdrawal. The participant, or the parent or person with legal responsibility for a child, will also be informed that they can choose to have their remaining blood samples destroyed and not stored for future analysis at the end of the trial.

The participant, or the parent or person with legal responsibility for a child, will be allowed time to consider the information, and the opportunity to question the Investigator or other independent parties to decide whether they will participate in the trial.

The participant, or parent or person with legal responsibility a child, must personally sign and date the latest approved version of the informed consent form. For children, where understanding is considered adequate, they will be asked to sign the assent form as per local ethics committee guidance. For children below the considered age, assent will be verbal, along with the consent from guardian. The person who obtained the consent must be suitably qualified and experienced, and have been authorised to do so by the Chief/Principal Investigator. A copy of the signed informed consent will be given to the participant. The original signed form will be retained at the trial site.

If the participant, or the parent or person with legal responsibility for a child, is illiterate then a third party independent of the study may act as a witness to attest that the information in the consent form and any other written information was accurately explained to, and apparently understood by, the participant, or the parent, or person with legal responsibility for a child, and that informed consent was freely given. In this event, the witness will also sign and date the consent form.

***Randomisation and treatment allocation.*** Participants will be randomly allocated to one of two treatment arms resulting in a 1:1 final disposition. The statistician in charge of randomization list preparation will set up statistical code to generate the randomization list and transfer it to the study pharmacist in accordance to the standard operation procedures (SOPs). The study pharmacist will then change the random seed, i.e. the initialization of the random numbers generator, in the statistical code in order to blind the biostatistician and then run the code to prepare the final randomization list. The generated randomization lists will be securely incorporated within the trial database. A reliable manual back-up system will also be available. The computer-generated randomisation list will use block randomization with stratification by site and age (children <16 years) and adults (≥16 years). The recruitment in most sites will take place during working hours as these are uncomplicated typhoid patients.

Randomisation will be performed using a web-based system accessed using an authorized username and password at each site and administered by the Oxford University Clinical Research Unit- Nepal Clinical Trial Unit independent from the study. Before treatment allocation the patient’s eligibility and informed signed consent will be confirmed and entered into the database. The allocation code will be held by the CTU pharmacist in Nepal.

### Study intervention

***Initial trial visit.*** Once consented, the following procedures will be completed and all details will be recorded in the case report form (CRF).

1. A member of the study team will collect demographic information (including age and address) and participant contact details.

2. A history and full physical examination will be performed. This will include current and past medical history and medications. Height/length and weight will be collected for all children <5 years of age.

3. Any additional laboratory samples will be collected and all available results will be reviewed ( Table 2: Schedule of events)

4. Upon successful randomisation, the clinician should then prescribe the trial drugs. Both the clinician and the patient will be blinded.

5. A study card containing the name of the study, and contact details for the study team will be given to the participant or the participant’s parents or person with legal responsibility to call if they have any concerns, or if admitted to hospital at any time during the duration of the study. The card will also contain instructions to attend the study clinic, if the participant becomes more unwell during the study period. Participants or participant’s parents or person with legal responsibility will be given details of the follow-up appointments.

**Table 2.** Schedule of events.

		Days following enrolment									
Study day	0	1	2	3	4	5	6	7	14	28	90 ^1^
Eligibility assessment	X										
Haematology(1mL)	X							(X) ^2^			
Biochemistry(1ml)	X							(X) ^2^			
Blood Culture (3/8ml)	X							X			
Blood for RDTs	X										
Blood for storage (1mL)	X									X	
Informed consent & patient information	X										
Urine stored antibiotic activity bioassay	X										
Stool culture/storage	X ^3^							X ^3^	X ^3^	X ^3^	(X) ^3^
Nasopharyngeal swab PCR/Antigen for COVID 19	X										
Randomization	X										
Drug Administration	X	X-X	X-X	X-X	X-X	X-X	X-X	X ^4^			
Hospital visits	X							X	X	X	(X)
CMA home visits			X		X						
Telephone calls	X	X-X	X	X-X	X	X-X	X-X				
Temperature *	X	X-X	X-X	X-X	X-X	X-X	X-X	X ^4^			
Adverse event assessment	X	X	X	X	X	X	X	X	X	X	

[ ^1^Followup on day 90 if the day 0 blood culture or faecal culture positive for *S*. Typhi or *S*. Paratyphi; ^2^Haematology and biochemistry repeated if day 0 results abnormal; ^3^Stool cultures for *S*. Typhi or *S*. Paratyphi on day 0, 7, 14, 28. Also on day 90 if the day 0 blood/stool culture positive for salmonella. ^4^If still febrile on day 7 twice daily assessments will continue until afebrile for48hrs and further treatment may be required as determined by the Trial Physician] PCR=polymerase chain reaction, COVID-19=coronavirus disease 2019, RDT=rapid diagnostic test. *37.5 degrees C axillary temperature.

***Follow-up trial visits.*** There will be routine participant follow-up via telephone or face-to face contact twice a day for the first seven days of antimicrobial treatment (and longer if the symptoms have not resolved). Caregivers are instructed to be consistent and measure temperature always at the same location (oral or axillary, not either or) for each patient and approximately at the same time. Face to face follow up by participant attendance at the clinic will be at 7 days, two weeks and one month. For patients who had positive blood or stool culture for *S.* Typhi or *S*. Paratyphi at the time of admission there will be a final clinic follow-up at three months.

The telephone calls will be made to ensure that clinical progress is satisfactory and for monitoring of symptoms. Any problems faced by participants can be discussed and possible adverse effects identified.

Community medical assistants (CMA) will visit the patients at their home on day 2 and day 4 of treatment to ensure that clinical progress is satisfactory and for monitoring. Special attention will be paid to the assessment of adherence to study medications, drug-related adverse events and disease related events. Adherence will be assessed using standardised questionnaires and pill counts. At the first visit their home location will be recorded using global positioning system (GPS).

All patients will return to the clinic for follow-up on day 7, day 14, day 28 (and day 90 if typhoid fever confirmed at onset) after the start of treatment for follow up clinical history, physical examination and sampling.

To ensure an optimal retention rate, the participants will be contacted by phone to remind them of their next visit. In addition, patients who have missed a visit will be contacted by phone for a maximum of three times after which a maximum of three home visits can be conducted. All contact attempts will be recorded.

### Laboratory testing and sample handling

Initial trial visit

- Ethylenediaminetetraacetic acid (EDTA) tube blood (1-1.5mL) for haematology (white cell count and differential, haemaglobin/haematocrit, platelet count) ( Table 2)
- Heparinised blood (2mL) for sodium, potassium, urea, creatinine, semi quantitative C reactive protein (CRP) test, bilirubin, aspartate transaminase (AST) and alanine transaminase (ALT)
- EDTA blood (2mL) will be used for malaria RDT, dengue RDT, scrub typhus RDT, and CRP RDT. Remaining blood centrifuged and plasma and blood pellet stored at -20°C /-80°C for future diagnostic, biomarker, pharmacokinetic studies and genetic analysis. Centrifugation and blood storage will be done ONLY if the patient fulfils the inclusion criteria.
- Blood culture (3-8mL depending on age).
- Urine sample will be collected from all participants and stored at -20°C/-80°C for later and tested by bioassay for antimicrobial activity.
- Faecal sample will be collected from all participants for standard bacterial culture (to detect *S*. Typhi or Paratyphi). DNA (deoxyribonucleic acid) will be extracted from the stool and stored for later diagnostic microbiome studies
- Nasopharyngeal swab test to detect COVID-19 using a PCR test or as per current existing rules and regulations of the hospital. Sample collection will be carried out following all the guidelines of the local hospital for handling potential COVID-19 patients.
- Rapid diagnostic tests: - For the malaria and dengue NS1 antigen RDTs locally available tests will be used. The scrub typhus RDT will be Scrub Typhus Detect™ IgM Rapid Test (InBios, Seattle, USA). For CRP screening we will use a semi-quantitative lateral flow test (ACTIM CRP tests, Medix Biochemica) that uses a drop of blood and gives a result in 5 minutes. It has thresholds of 10, 40 and 80mg/L so will give the right cutoff for inclusion, and some information on how high CRP is when it is ≥10mg/L.

Follow up trial visits

A blood culture will be repeated at the day 7 follow up visit and at any time during follow-up if there is a clinical deterioration in the participant or a clinical suspicion of relapse after review and assessment by the trial physician. Stool cultures for *S*. Typhi or *S.* Paratyphi on day 0, 7, 14, 28. Also on day 90 if the day 0 blood/stool culture positive for salmonella.

### General comments

Blood samples drawn in the study clinic for diagnosis and confirmation of suspected typhoid fever will be handled, stored, processed, in accordance with standard operating procedures of the study Microbiology Laboratory. The results of these tests will be recorded in the participant CRF for use in this study.

Remaining samples will be stored at the local laboratory for further analysis, depending on funding availability, after the conclusion of the trial. This analysis will include diagnostic testing (serology or PCR) to determine additional causes of infection in the blood culture negative group.

Additionally DNA extraction for investigation of the genetic control of susceptibility to infectious diseases like typhoid and pharmacogenetic determinants may be carried out. DNA will be genotyped/sequenced and analysis will be performed (genome wide association study or GWAS analysis, and DNA sequence analysis).

At selected sites, additional samples will be taken for pharmacokinetic analysis. This will be one or two additional EDTA samples taken during the course of treatment. The cells and plasma will be separated and stored at -20°C/-80°C. These samples will subsequently be assayed for azithromycin and/or cefixime using high-performance liquid chromatography (HPLC). The data will be analysed using a population pharmacokinetic modelling approach using Pharmacokinetic/Pharmacodynamic Systems Analysis Software ADAPT5.

Bacterial isolates will be stored at -20°C/-80°C for later whole genome sequencing. The WGS will be performed at study sites if they have capacity, or at the Wellcome Sanger Institute, UK. This will be to determine if recurrent positive blood cultures are due to relapse or re-infection, to understand the population structure of *S*. Typhi/ *S.* Paratyphi across South Asia and characterise relevant resistance and/or virulence mechanisms.

### Sample size calculation

We will randomize 1500 subjects (750 per arm) within 2 years, with between 125 and 250 recruited per year at each site. Assuming a treatment failure risk of 15% in the group given azithromycin alone, the sample size will guarantee 92% power to detect an absolute risk reduction of 6% (from 15% to 9%) and 80% power to detect an absolute risk reduction of 5% (from 15% to 10%) in the group given the combined antimicrobials at the two-sided significance level of 5% allowing for up to 10% loss to follow up. In case of a lower treatment failure risk of 10% in the control arm, the trial will have 94% power to detect a 5% absolute risk reduction (from 10% to 5%) and 77% to detect a 4% reduction (from 10% to 6%).

### Statistical and analytical plans

The statistical aspects of the study are summarised here with details fully described in a statistical analysis plan (SAP) which will be finalised before any analysis takes place. The primary endpoint of this trial is the composite outcome of treatment failure by the 28th day after the initiation of treatment. Subjects who withdraw from the study prior to day 28 without treatment failure will be treated as right-censored. The primary comparison of the absolute risk of treatment failure until day 28 between the treatment arms will be based on Kaplan-Meier estimates and corresponding standard errors according to Greenwood’s formula.

The time to treatment failure from the onset of treatment will be described using Kaplan-Meier curves and compared between the treatment arms with a Cox proportional hazards model with treatment assignment as the primary covariate and adjustment for blood culture result (positive for *S.* Typhi or *S.* Paratyphi vs. negative), age (children (<16 years) and adults (≥16 years)), and country. Time to treatment failure from symptom onset will be analysed in the same way but subjects will be considered as left-truncated at the time of onset of treatment to account for the fact that they were not under observation before that timepoint.

Fever clearance time will be calculated using temperatures recorded twice per day and treated as an interval-censored outcome. The fever clearance time distribution will be estimated using the non-parametric maximum likelihood estimator (NPMLE) and comparisons between the arms will be based on a parametric Weibull accelerated failure time model. 

A two-sided significance level of 5% will be applied to all efficacy analyses. The primary analysis population for all analyses is the modified intention to treat (ITT) population consisting of all patients who have been randomised to the trial and received at least one dose of study treatment. Analysis will be according to the randomized treatment arm. Subjects, who were mistakenly randomised or withdrew before the first dose of study treatment was given, will be excluded. Subjects who were unblinded will be included in the ITT population.

The culture-confirmed population consists of all patients with blood-culture confirmed typhoid fever who received at least one dose of study treatment. Analysis will be according to the randomized treatment arm. The primary and secondary endpoints will be analysed both in the ITT and the culture-confirmed population. Planned sub-group analyses for the primary endpoint and fever clearance time include analyses by blood culture result (positive for *S.* Typhi or *S.* Paratyphi vs. negative), by age (children (<16 years) and adults (≥16 years)), by country, and by study site. 

A safety interim analysis will be conducted at 6 months and a combined safety and efficacy interim analysis will be conducted at 50% sample size, i.e. when outcome data from 750 subjects are available. Formal stopping for efficacy is only foreseen at the efficacy interim analysis in case of overwhelming efficacy for the combined treatment using the Haybittle-Peto boundary (p<0.001) as a guidance.

The efficacy interim analysis will also include a futility analysis which will assess the conditional power to detect a 6% difference in failure rates at the final analysis. If the conditional power is low, the DSMC may recommend closing the trial for futility or increasing its sample size. The final decision making in case of a sample size increase will require input from the Trial Steering Committee, the JGHT Committee and the joint funders. All analyses will be done with R language for statistical computing version 3.6.2 software.

### Data collection and management

Data collection is the responsibility of the clinical trial staff at the site under the supervision of the site PI. The investigator is responsible for ensuring the accuracy, completeness, legibility, and timeliness of the data reported. Clinical data (including adverse events (AEs), concomitant medications, and expected adverse reactions data) and clinical laboratory data will be entered into CliRes, a 21 CFR Part 11-compliant data capture system provided by the OUCRU IT department. The data system includes password protection and internal quality checks, such as automatic range checks, to identify data that appear inconsistent, incomplete, or inaccurate. Clinical data will be entered directly in tablets will be utilized from the various sites to enter data directly to the central database collection site at the Nepal CTU. The sites can access their data in the central data base which will be password protected.

The primary data is collected onto the CRF while interviewing the patients at the outpatient department in the hospital. There will be 1500 patients, with 5 hospital visits each, 2 home visits by CMA and data obtained from the telephone calls. On average, each patient will have a CRF, which contains around 23–25 pages with all patient details. The participants will be identified by a unique trial specific number and/or code in any database. The name and any other identifying detail will NOT be included in any trial data electronic file.

Only authorized users with specific delegation will be given access to the database. These users will be trained according to their delegation before working with the database. All the changes made in the database will be tracked and will be kept as a part of documentation. Data storage will be in the central database in the Nepal CTU and backed up in Vietnam. All the sites will be able to access their data secured with password from the central site. All the metadata will be specified during the database development which includes data type, coding, table and column names, validation etc. The data required for the analysis from the extracted database will be derived from the metadata dictionary.

### Data preservation strategy and standards

All the physical data from the CRFs will be collected in an electronic tablet in each site which will feed into the central electronic data base which will be stored securely at the OUCRU Nepal server with regular backup in Vietnam. The data security standards in place at each site are detailed in the unit’s system level security policy which adheres to the standard for information security management ISO27001. All the trials participants’ anonymity and confidentiality will be maintained throughout the study period. The same will be ensured with the data of the participants. They will be identified by the specific study numbers rather than their name or any other identifiable characters. All the users involved in the data management will be trained before allocation to the specific tasks related to patient details.

### Data sharing and access

Access to data for outside party will be given only after approval by the Trial steering group which will be through application to the group. OUCRU CTU SOP ensures participants’ anonymity and Confidentiality during the data sharing procedure. The Nepal CTU will ensure the trial is `discoverable’ through trial registration.

### Safety reporting

The definitions of the EU Directive 2001/20/EC Article 2 based on the principles of the International Conference on Harmonization good clinical practice (ICH GCP) apply to this trial protocol. Adverse effects will be classified and graded according to the Common Terminology Criteria for Adverse Events (CTCAE) system. All serious and grade 3 or 4 AEs will be compared between arms and reported by frequency per arm. An independent DMC will oversee the safety of the trial participants Definitions. The definitions of the principles of ICH GCP apply to this trial protocol. All grade 3 or 4, or serious AEs and ARs, whether expected or not, should be recorded in the CRF. Non-serious grade 1 or 2 AEs need not be recorded unless they are thought to be related to the IMP or they result in a change or interruption in treatment.

Procedure for immediate reporting of serious adverse events (SAEs)

- Site study team will complete an SAE report form for all reportable SAEs.
- Where the SAE requires immediate reporting, the SAE report form will be scanned and emailed to trial coordinating team immediately i.e., within 24 hours of site study team becoming aware of the event. The team will acknowledge receipt of this report.
- Site study team will provide additional, missing or follow up information in a timely fashion.
- The assessment of expectedness will be conducted within 48 hours by the Site study team and the Coordinating Centre team within three days of reporting.
- All deaths will be reported to the trial coordinating team immediately i.e., within 24 hours of site study team becoming aware of the event and to the chairman of the Data Safety Monitoring Board (DSMB)

All SAEs other than those defined in the protocol as not requiring reporting must be reported on the SAE reporting form to CTU within 24 hours of the trial coordinating team becoming aware of the event. 

SAEs will be reported as soon as possible to the site ethics committee (EC). A written report will be sent as soon as possible but not later than 2 working days (for SAE resulting in death or life threatening). Additionally, the detailed report of the SAE should be submitted to the IRBs within 15 days. Additional medical information of the SAE’s development must be reported in other report until the trial subjects recover or stabilize without further change expected. Other AEs should be recorded, summarized and reported in the annual report form and the final report form. All SAEs will be reported to OxTREC in the annual review form and to the DMC in accordance to the DMC charter.

### Risk assessment

The trial will be conducted in accordance with the current approved protocol, GCP, relevant regulations and standard operating procedures including precautions regarding COVID-19. A risk assessment and monitoring plan will be prepared before the study starts and will be reviewed as necessary over the course of the trial to reflect significant changes to the protocol or outcomes of monitoring activities.

### Monitoring

Regular monitoring will be performed according to the trial specific monitoring plan. Data will be evaluated for compliance with the protocol and accuracy in relation to source documents as these are defined in the trial specific monitoring plan. Following written standard operating procedures, the monitors will verify that the clinical trial is conducted and data are generated, documented and reported in compliance with the protocol, GCP and the applicable regulatory requirements.

### Ethical and regulatory considerations

The Investigator will ensure that this trial is conducted in accordance with the principles of the Declaration of Helsinki and with relevant regulations, approved protocol and good clinical practice. Following Sponsor approval, the protocol, informed consent form, participant information sheet and proposed advertising material has been approved by the Oxford Tropical Research Ethics Committee (OXTREC) (reference number 28-20) and the Nepal Health Research Council (reference number 2291). The decision from the remaining review boards is pending.

The rights of the participant to refuse to participate in the trial without giving a reason must be respected. After the participant has entered into the trial, the clinician must remain free to give alternative treatment to that specified in the protocol, at any stage, if he/she feels it to be in the best interest of the participant. The reason for doing so, however, will be recorded; the participant will remain within the trial for the purpose of follow-up and for data analysis by the treatment option to which they have been allocated. Similarly, the participant must remain free to change their mind at any time about the protocol treatment and trial follow-up without giving a reason and without prejudicing his/her further treatment.

All participants will receive the best available treatment of typhoid fever, following local and national guidelines. They will benefit from the frequent and careful follow-up of their condition throughout the treatment of their typhoid fever and for up to 28 days from randomization.

The risks of participation are few. Azithromycin and cefixime are commonly prescribed drugs and there is widespread experience and expertise concerning their safe use. The choice dose, route of administration and duration of study treatment follows the international guidance for the treatment of typhoid fever. Previous trials have demonstrated the safety of this or similar regimens.

Against these minimal risks, trial participants may benefit from receiving these antibiotics for treatment of their suspected or confirmed typhoid fever. In addition, all participants will benefit from the careful observation and follow-up over 28 days from enrolment, which will allow complications of typhoid fever to be rapidly identified and managed.

The risks and benefits of participation will be communicated in two ways. First, all potential participants or their family members will be given a participant information sheet clearly listing the risks and benefits of the trial. Second, all potential participants (or their families) will be able to discuss participation with their consulting doctor who will be able to address questions not covered or arising from the participant information sheet.

Participants’ confidentiality will be maintained throughout the trial. Data submitted to Nepal CTU and samples sent to central testing facilities will be identified only by the trial number and participant initials.

The Principal Investigator shall submit once a year throughout the clinical trial, or on request, an annual progress report to the REC, HRA (where required), host organisation, funder (where required) and Sponsor. In addition, an End of Trial notification and final report will be submitted to the local and national ethics committee, host organisation and Sponsor within 12 months of completion of the study.

The study will comply with the General Data Protection Regulation (GDPR) and Data Protection Act 2018, which require data to be de-identified as soon as it is practical to do so. The processing of the personal data of participants will be minimised by making use of a unique participant study number only on all study documents and any electronic database(s), with the exception of the CRF, where participant initials may be added. All documents will be stored securely and only accessible by study staff and authorised personnel. The study staff will safeguard the privacy of participants’ personal data.

### Expenses and benefits

Reasonable travel expenses for any visits additional to normal care will be reimbursed adequately to cover their costs on production of receipts, or a mileage allowance will be provided as appropriate. In addition, the cost of trial-related AEs that lead to hospital admission or treatment will be covered by the study

### Finance and Insurance

***Insurance.*** The University has a specialist insurance policy in place which would operate in the event of any participant suffering harm as a result of their involvement in the research (Newline Underwriting Management Ltd, at Lloyd’s of London).

***Contractual arrangements.*** Appropriate contractual arrangements will be put in place between the University of Oxford and each site involved in the study.

### Publication policy

The investigators will co-ordinate dissemination of data from this study. For dissemination of research, we will give presentations in typhoid meetings in Nepal, India, Bangladesh, Pakistan and in international forums like The American Society of Tropical Medicine and Hygiene (ASTMH), The Royal Society of Tropical Medicine and Hygiene (RSTMH) and also in infectious disease societies in each of the collaborator countries. We will also engage with local World Health Organization (WHO) and health ministries to see if the findings from the study can have impact on policy. Finally, we hope to publish the findings in a weekly international medical journal for a wide impact. All publications, including manuscripts, abstracts, oral/slide presentations, and book chapters, etc., based on data from this study will be reviewed by each sub-investigator prior to submission. Authors will acknowledge that the study was funded by MRC. In accordance with MRC, all publications related to this study will be open access. Authorship will be determined in accordance with the International Committee of Medical Journal Editors (ICMJE) guidelines and other contributors will be acknowledged.

### Archiving

Study documents (both original and electronic version) will be maintained for time specified by the study protocol, sponsor and regulatory authority. Data will be stored in centralised Oxford University Clinical Research Unit (OUCRU) server which is backed up regularly. These documents will be archived in the study site for defined retention period securely. No personal identifiers or participants information will be included in the archive.

## Discussion

In this study, we will conduct a randomised comparison of azithromycin alone or azithromycin combined with cefixime for treating clinically suspected or confirmed uncomplicated typhoid fever at four sites across south Asia. We aim to test the hypothesis that the treatment failure proportion will be 15% with azithromycin alone but 9% if the azithromycin is combined with cefixime. When translated to the number of patients with typhoid fever across south Asia this improvement in outcome would make a substantial real-world contribution to improving the health of the population in our region. With an estimated 7 million people suffering from typhoid fever in South Asia each year ^1^, a 6% reduction in treatment failures in this population using this combination of drugs will mean at least 420,000 patients avoiding the need for further antimicrobial treatment and/or hospital admission, and consequently result in a lower financial burden and a reduced potential for onward transmission of the infection.

Cefixime and azithromycin are widely used antimicrobials in typhoid fever with excellent safety profiles. If the combination treatment is better than the single antimicrobial treatment, this will be an important result for patients across South Asia and other typhoid endemic areas. We will also additionally investigate the financial implications for families and health system. Combined treatment may also limit the emergence of resistance if one of the components is active against resistant sub-populations not covered by the other antimicrobial’s activity. This is part of the rationale for using combination chemotherapy in other infections such as malaria, TB and HIV. In *S.* Typhi and *S.* Paratyphi A there are sporadic reports of resistance to both azithromycin and third generation cephalosporins ^33, 34^. The recent outbreak of MDR, fluoroquinolone and ceftriaxone resistant (XDR) typhoid in Pakistan is a warning of the potential for untreatable typhoid ^35^. Azithromycin resistance in enteric fever pathogens would leave a major gap in treatment options. This combination would still be efficacious if the infecting pathogen was resistant to one of the drugs. If it could delay or prevent the emergence of this resistance that would have an important public health benefit. Finally, combination treatment may reduce the emergence of resistance in Enterobacteriaceae in gastrointestinal microbiota. As the use of drug combinations would lead to increased costs and more potential side-effects it is critically important to establish if there is a measurable clinical benefit of combining these antimicrobials. This potential effect is particularly important as fixed dose combinations of these antimicrobials are already available despite government regulations in India ^36^. Hence even if this study shows that the combined therapy has no advantage over a single drug treatment for suspected enteric fever, it will be important to know this against the background of the availability and anecdotal usage of the combination.

There are two published systematic reviews that address the antimicrobial treatment of typhoid fever. One review ^11^ focusing on the fluoroquinolones concluded that this class of antimicrobial performed well in treating typhoid but that clinicians needed to take into account local resistance patterns. Recent data ^2^ suggests that across most of South Asia the levels of non-susceptibity to fluoroquinolones is too high for this to be a reliable treatment option. The second systematic review focused on the role of azithromycin ^7^, in which seven RCTs involving 773 patients were identified. In comparison to the older fluoroquinolones (213 participants), there were fewer clinical failures (RR 0.46 (0.25-0.82)) while in comparison with ceftriaxone (132 participants) there was a significant reduction in the chance of relapse (RR 0.1 (0.01-0.76)). Both these reviews noted that most trials had been conducted on in-patients and may not therefore be representative of settings where most enteric fever is managed as an out-patient. There were also few studies in each comparison with studies that were too small to make firm conclusions on the presence or absence of important differences. The authors recommended a need for multi-centred, adequately powered trials with robust methods and analytical design. Many RCTs have only analysed the culture positive cases rather than ‘intention-to-treat ‘. The ACT-South Asia attempts to address these points by conducting a large multi-centre study of the out-patient treatment of uncomplicated typhoid fever in the largest hub for typhoid fever in the world.

The outcome of this first major regional collaborative RCT designed to deal with a rampant scourge will be of crucial importance for clinicians working in South Asia as it may help to guide the best empirical treatment for suspected typhoid fever and reduce the rates of treatment failure, the risk of complications and hospital admission, the overuse of more expensive second line antimicrobials and extra healthcare costs. The appearance of XDR typhoid in Pakistan ^3^ is a pressing reason why this trial is needed now as there is clearly a risk of these XDR organisms spreading to other highly endemic sites in South Asia or for such organisms to emerge in other endemic locations. This study is of relevance to all of the proposed study sites as healthcare workers at each site contend with the management of patients with typhoid fever on a daily basis. Including sites from across the sub-continent will make the results generalisable to other locations in South Asia where similar problems with typhoid fever are found ^37^. Most typhoid fever in sub-Saharan Africa currently still responds to fluoroquinolone treatment but reports of fluoroquinolone resistance are emerging and alternative regimens will be needed if resistance become widespread. Finally, in our analysis, the effect of the intervention in the culture-negative patients may provide useful data to inform empiric treatment algorithms for both blood culture negative acute febrile illness in this region ^38^ and also for medical facilities in vast swathes of South Asia where blood cultures are unavailable.

### Study status

Recruitment was planned to start from September 2020. However, study initiation has been significantly affected by the pandemic all sites. Nepal has started patient enrolment and first patient was enrolled on 23 May 2021. Nine patients have been recruited in the trial already. Pakistan will probably be the next site to start enrolment soon followed by the other sites.

## Data availability

### Underlying data

No data are associated with this article.

### Extended data

Oxford University Research Archive (ORA): Dataset for ACT-South Asia protocol manuscript. https://doi.org/10.5287/bodleian:OBopRno5q ^29^.

This project contains the following extended data:

- DSMC charter for ACT South Asia_V1.pdf (Data & Safety Monitoring Committee)

- PIS_informed_consent_adults.pdf (Patient information sheet and consent form for adults)

- Drug_dosing_table-ACT South Asia.pdf (Drug dosing table)

- PIS_and _assent form.pdf (Patient information sheet and assent form for children)

### Reporting guidelines

Oxford University Research Archive (ORA): SPIRIT checklist for ‘Azithromycin and cefixime combination versus azithromycin alone for the out-patient treatment of clinically suspected or confirmed uncomplicated typhoid fever in South Asia; a randomised controlled trial https://doi.org/10.5287/bodleian:OBopRno5q ^29^.

Data are available under the terms of the Creative Commons Attribution 4.0 International license (CC-BY 4.0).
