# Supplementary material for: Azithromycin and cefixime combination versus azithromycin alone for the out-patient treatment of clinically suspected or confirmed uncomplicated typhoid fever in South Asia: a randomised controlled trial protocol
Source: Wellcome Open Res. 2021 Nov 12;6:207. Originally published 2021 Aug 11. [Version 2] doi: 10.12688/wellcomeopenres.16801.2 (PMC8772527; doi:10.12688/wellcomeopenres.16801.2)
Supplement: Peer review history

# Peer review: Reviewer response for version 2

**DOI:** 10.21956/wellcomeopenres.19206.r47062

**Published:** 2021-12-08

**Authors:** Eli Schwartz

**Affiliations:** 1 The Center for Geographic Medicine and Tropical Diseases, Sheba Medical Center, Ramat Gan, 52621, Israel

The primary analysis of this study is the intention to treat, including those with culture negative. This group of patients on one hand represents the real group which is seen in the clinics in daily life. But, on the other hand , a major part of them might have  another (bacterial most likely) infections. Therefore all efforts should be done to get an accurate diagnosis of those who were culture negative. In this regard:

1. What about those who will be found to have another diagnosis, are they included in the ITT analysis?
2. As I mentioned previously one of the common febrile illnesses in these regions (mainly in  agriculture population) is **Leptospirosis **which is  a neglected disease (probably due to lack of  diagnostic methods). More efforts should be carried on to identify it. Since blood (serum ) is taken on day 0 and day 7, that sample can be kept for all kinds of serology including leptospirosis.

Is the study design appropriate for the research question?

Partly

Is the rationale for, and objectives of, the study clearly described?

Yes

Are sufficient details of the methods provided to allow replication by others?

No

Are the datasets clearly presented in a useable and accessible format?

Yes

Reviewer Expertise:

NA

I confirm that I have read this submission and believe that I have an appropriate level of expertise to confirm that it is of an acceptable scientific standard.

# Response

**Published:** 2021-12-11

**Authors:** Buddha Basnyat

**Affiliations:** OUCRU, Nepal

Thank you very much, Dr Eli Schwartz. 

 In answer to your first query, yes indeed those that are found to have another diagnosis will be included in the ITT analysis. And we will also have a sub-group analysis of just those who are culture positive for typhoid fever. So, this will be a wide-ranging study the results of which should impact the treatment of undifferentiated febrile illness in South Asia including typhoid fever.

 In response to your second query regarding serological diagnosis of leptospirosis and other common ( often neglected) disease-causing organisms, we are taking this matter up seriously and actively looking for means to carry out these tests.

# Peer review: Reviewer response for version 2

**DOI:** 10.21956/wellcomeopenres.19206.r47063

**Published:** 2021-11-16

**Authors:** Karin Leder

**Affiliations:** 1 School of Public Health and Preventive Medicine, Monash University, Clayton, Vic, Australia

Thank you for the opportunity to review the authors responses to reviewer comments, which are clear and sufficiently detailed.

Is the study design appropriate for the research question?

Partly

Is the rationale for, and objectives of, the study clearly described?

Yes

Are sufficient details of the methods provided to allow replication by others?

Yes

Are the datasets clearly presented in a useable and accessible format?

Not applicable

Reviewer Expertise:

Infectious diseases, study design

I confirm that I have read this submission and believe that I have an appropriate level of expertise to confirm that it is of an acceptable scientific standard.

# Peer review: Reviewer response for version 1

**DOI:** 10.21956/wellcomeopenres.18533.r45461

**Published:** 2021-09-23

**Authors:** Eli Schwartz

**Affiliations:** 1 The Center for Geographic Medicine and Tropical Diseases, Sheba Medical Center, Ramat Gan, 52621, Israel

This is an extremely important study which could have an important impact globally on the approach for treating people with typhoid. It could also have an economic impact as well as possibly contribute to changes in the epidemiology of the disease (see below). It is unfortunate that this trial took such a long time to be conducted.

 From an historical perspective, it is interesting and important to elaborate on the role of the field of travel medicine in the development of this therapeutic solution.

 Treating enteric fever (typhoid and paratyphoid fever) became a challenge during the two decades when resistance to quinolones, the magic bullet against typhoid fever, became evident. Interestingly, the call for a new approach was declared first from the Travel Medicine practitioners who reported  the unsatisfactory response to both  3 ^rd^ generation cephalosporins (despite being  sensitive in-vitro) and to azithromycin when each of them was given alone. The proposed approach in 2009 ^1^ was to give these two drugs, azithromycin and 3 ^rd^ generation intravenous (IV) cephalosporins, together. The idea was that these two drugs would cover the extracellular and intracellular compartments, the two compartments where the typhoid bacteria is shifting between. The high efficacy of fluoroquinolones before the development of NA-resistant strains can perhaps be attributed to their excellent distribution in both the intra- and extracellular compartments.

 This proposal was introduced as the novel treatment for typhoid fever and became the policy in Sheba Medical Ctr. In Israel. It was further approved during a major paratyphi outbreak in Israeli travelers in Nepal, reported in 2014. ^2^ In this observational study, combined therapy shortened the febrile days from 6 to 3 days. This reference somehow was omitted in the current proposal by Abhishek Giri, *et al.*

 The Israeli concept was further tested in a comparative study done among local populations in Nepal [appears as Ref# 17 in the proposal]. In this trial, patients with typhoid and paratyphoid fever were included (not only paratyphi, as was observed in the previous study). The other advantage of the Nepalese study was the inclusion of an oral cephalosporin arm in non-hospitalized patients (and not only IV ceftriaxone) which is appropriate in the environment of most of the endemic countries where the majority of patients are treated as outpatients. The results showed that the combination of third-generation cephalosporins and azithromycin may confer a more effective therapy, reducing the time to defervescence and to the clearance of bacteremia.

 The current large scale, multi-country trial, as proposed now by the team, is most welcome. The design of the study is rigorous, including a placebo arm and a much larger number of participants. As the author mentioned, successful results will certainly lead to an adoption of this policy and bring with it great benefits to the patients as well as have a positive impact on the economy.

 An interesting outcome which might be monitored using the combined therapy is whether we will also see a  decrease in the fecal-carriage rates (we in fact do not see fecal-carriage in our travelers, unfortunately the Nepalese study#17 was too small to detect it) and in this case the spread of the disease might be limited, having an important epidemiological impact. This aspect should be highlighted in the manuscript.

 Other aspects:

- Since the absorption of oral cephlosporins is not steady, results might be different from IV ceftriaxone treatment. I wonder whether in this huge trial a small fraction of hospitalized patients can be recruited to test the combination of IV ceftriaxone + azithro against one of the drugs + placebo. (I would guess ceftriaxone + oral placebo).

- It is not clear (or perhaps I missed it) whether those with negative cultures will be included in final analysis. If yes, what about all those who subsequently will have  (with the more sophisticated tests) another microbial diagnosis?

- There are lists of rapid tests that will be performed; one of the common febrile illnesses in these regions is **Leptospirosis **which is not mentioned on the list. Should a rapid test or PCR be added to the list?
- Blood culture- As I know there are different blood-culture bottles in these countries with different amount of blood, which gives different sensitivity.  Are these centers going to be supplied from a central supplier? There is no clear instruction about the amount of blood.
- The Placebo mentioned is sugar pills. I am concerned that this may prevent a real blinding of the study.
- There is redundancy in secondary end-point and the statement below appears 3 times.

*•* *To compare the time from onset of treatment to treatment failure in patients in each treatment arm*

*•* *To compare the time from onset of symptoms to treatment failure in patients in each treatment arm*

*•* *To compare the time from onset of symptoms to treatment failure in patients in each treatment arm*

  -

**Ref**

1. Schwartz E. Typhoid and paratyphoid fever. In: Schwartz E, editor. Tropical Diseases in Travelers. New York, NY: John Wiley & Sons; 2009: 144-153.
2. Meltzer E, Stienlauf S, Leshem E, Sidi Y, Schwartz E. A large outbreak of Salmonella Paratyphi A infection among israeli travelers to Nepal. Clin Infect Dis. 2014 Feb;58(3):359-64. doi: 10.1093/cid/cit723. Epub 2013 Nov 5. PMID: 24198224.

Is the study design appropriate for the research question?

Partly

Is the rationale for, and objectives of, the study clearly described?

Yes

Are sufficient details of the methods provided to allow replication by others?

No

Are the datasets clearly presented in a useable and accessible format?

Yes

Reviewer Expertise:

Travel and Tropical Medicine

I confirm that I have read this submission and believe that I have an appropriate level of expertise to confirm that it is of an acceptable scientific standard, however I have significant reservations, as outlined above.

## References

[1] : Typhoid and paratyphoid fever in E. Schwartz (ed.) Tropical Diseases in Travelers. *John Wiley* .2009;144-153

[2] : A large outbreak of Salmonella Paratyphi A infection among israeli travelers to Nepal. *Clin Infect Dis* .2014;58(3) : 10.1093/cid/cit723 359-64 10.1093/cid/cit723 24198224

# Response

**Published:** 2021-11-08

**Authors:** Abhishek Giri

**Affiliations:** Oucru-Nepal, Nepal

We would like to thank Eli Schwartz for his comprehensive input that has definitely enhanced the presentation of the protocol in its clarity and focus.

 Below is our point-by-point response to both the Reviewers and suitable changes have also been made in the track-changed manuscript. Reviewer comments in italics.

***Comments***

*This is an extremely important study which could have an important impact globally on the approach for treating people with typhoid. It could also have an economic impact as well as possibly contribute to changes in the epidemiology of the disease (see below). It is unfortunate that this trial took such a long time to be conducted.*

*From an historical perspective, it is interesting and important to elaborate on the role of the field of travel medicine in the development of this therapeutic solution.*

*Treating enteric fever (typhoid and paratyphoid fever) became a challenge during the two decades when resistance to quinolones, the magic bullet against typhoid fever, became evident. Interestingly, the call for a new approach was declared first from the Travel Medicine practitioners who reported  the unsatisfactory response to both  3 ^rd^ generation cephalosporins (despite being  sensitive in-vitro) and to azithromycin when each of them was given alone. The proposed approach in 2009 ^1^ was to give these two drugs, azithromycin and 3 ^rd^ generation intravenous (IV) cephalosporins, together. The idea was that these two drugs would cover the extracellular and intracellular compartments, the two compartments where the typhoid bacteria is shifting between. The high efficacy of fluoroquinolones before the development of NA-resistant strains can perhaps be attributed to their excellent distribution in both the intra- and extracellular compartments.*

*This proposal was introduced as the novel treatment for typhoid fever and became the policy in Sheba Medical Ctr. In Israel. It was further approved during a major paratyphi outbreak in Israeli travelers in Nepal, reported in 2014. ^2^ In this observational study, combined therapy shortened the febrile days from 6 to 3 days. This reference somehow was omitted in the current proposal by Abhishek Giri, et al.*

*The Israeli concept was further tested in a comparative study done among local populations in Nepal [appears as Ref# 17 in the proposal]. In this trial, patients with typhoid and paratyphoid fever were included (not only paratyphi, as was observed in the previous study). The other advantage of the Nepalese study was the inclusion of an oral cephalosporin arm in non-hospitalized patients (and not only IV ceftriaxone) which is appropriate in the environment of most of the endemic countries where the majority of patients are treated as outpatients. The results showed that the combination of third-generation cephalosporins and azithromycin may confer a more effective therapy, reducing the time to defervescence and to the clearance of bacteremia.*

*The current large scale, multi-country trial, as proposed now by the team, is most welcome. The design of the study is rigorous, including a placebo arm and a much larger number of participants. As the author mentioned, successful results will certainly lead to an adoption of this policy and bring with it great benefits to the patients as well as have a positive impact on the economy.*

*An interesting outcome which might be monitored using the combined therapy is whether we will also see a  decrease in the fecal-carriage rates (we in fact do not see fecal-carriage in our travelers, unfortunately the Nepalese study#17 was too small to detect it) and in this case the spread of the disease might be limited, having an important epidemiological impact. This aspect should be highlighted in the manuscript.*

*Other aspects:*

- *Since the absorption of oral cephlosporins is not steady, results might be different from IV ceftriaxone treatment. I wonder whether in this huge trial a small fraction of hospitalized patients can be recruited to test the combination of IV ceftriaxone + azithro against one of the drugs + placebo. (I would guess ceftriaxone + oral placebo).*

* It is not clear (or perhaps I missed it) whether those with negative cultures will be included in final analysis. If yes, what about all those who subsequently will have  (with the more sophisticated tests) another microbial diagnosis?*

**Response**

 We thank the Reviewer for these very important comments emphasizing the points that have been raised in the protocol. Indeed studies (in Nepalis with typhoid fever from Dhulikhel hospital ( ref 17) and returned travelers from Nepal published in the CID in 2014) carried out by Dr. Eli Schwartz and his team actually helped trigger the concept for this proposal. We mistakenly referenced another article instead of the correct CID article by Meltzer et al ( # 28). The mistake has now been rectified and we thank the reviewer for pointing this out.

 We have also now further emphasized at the end of the manuscript the fact that if the combination treatment arm results in the less fecal carriage, this will clearly be helpful in the control of the spread of the disease by carriers.

 Regarding the other Aspects portion,  testing ceftriaxone will not be possible as this is a study of outpatients, not hospitalized patients, as mentioned by the Reviewer.

 Finally, we are indeed trying to obtain funding to carry out studies in the culture-negative group to ascertain etiology.

# Response

**Published:** 2021-11-24

**Authors:** Buddha Basnyat

**Affiliations:** OUCRU, Nepal

**We beg Reviewer two's pardon in that we missed responding to his last 4 important queries. Our responses are in bold and the reviewers comments are in italics.**

*There are lists of rapid tests that will be performed; one of the common febrile illnesses in these regions is Leptospirosis ****which is not mentioned on the list. Should a rapid test or PCR be added to the list?*

**The reviewer raises an important point but** t **here are no rapid reliable tests including PCR available for us at this stage to include this in the protocol.**

*Blood culture- As I know there are different blood-culture bottles in these countries with different amount of blood, which gives different sensitivity.  Are these centers going to be supplied from a central supplier? There is no clear instruction about the amount of blood.*

**Under lab testing we have indeed mentioned in the protocol that 3 to 8 ml will be sampled from the patient depending on the age of the patient (and BACTEC bottles will be used in all sites). There will be no central supplier.**

*The Placebo mentioned is sugar pills. I am concerned that this may prevent a real blinding of the study.* 

**We have tasted both the placebo and the real cefixime and they taste no different.**

*There is redundancy in secondary end-point and the statement below appears 3 times.* 

- *To compare the time from onset of treatment to treatment failure in patients in each treatment arm*
- *To compare the time from onset of symptoms to treatment failure in patients in each treatment arm*
- *To compare the time from onset of symptoms to treatment failure in patients in each treatment arm*   

**We did not see this redundancy in the Secondary Endpoints. Perhaps the Reviewer was referring to Secondary Objectives. But again there is no redundancy because we are trying to compare onset of symptoms as well as onset of treatment separately to treatment failure.**

**Thank you very much for your kind consideration.**

**Dr Basnyat, PI**

# Peer review: Reviewer response for version 1

**DOI:** 10.21956/wellcomeopenres.18533.r45458

**Published:** 2021-08-25

**Authors:** Karin Leder

**Affiliations:** 1 School of Public Health and Preventive Medicine, Monash University, Clayton, Vic, Australia

**Main comments**

 The rationale for performing this study to assess the efficacy of monotherapy versus combined therapy for treatment of typhoid fever is well-justified, and the protocol is generally clear and sufficiently detailed.

 However, I have suggested that the study design is only partly appropriate for the research question because patients with suspected typhoid fever will be included, thereby mixing outcomes of patients with typhoid and those with other conditions. I note that the main analysis will be as ITT, but that primary and secondary endpoints will be analysed both as ITT and among the culture-confirmed population. I realise that the ITT design has practical and logistic advantages, but some non-typhoidal bacterial infections will also respond to these antibiotics. Consequently, since the main analysis is ITT, there is an apparent mismatch with the stated key primary outcome – to determine whether combination therapy is superior to azithromycin minor in preventing treatment failure in patients with uncomplicated typhoid fever. In fact, the ITT analysis will answer whether combination therapy is superior to azithromycin minor in preventing treatment failure in patients with suspected typhoid fever, but not specifically in those with uncomplicated typhoid fever. Both questions have clinical importance, but the precise main research question being addressed should be accurately stated, and should be directly matched to the inclusion criteria and the primary endpoints (currently a mix of clinical and typhoid-specific microbiological criteria).

 My concern about the precise research question also relate to the power calculations, where there is a lack of clarity regarding whether it is suspected or proven infection that serves as the main endpoint. No data are provided on the proportion of suspected cases that – after exclusion of malaria, dengue, scrub typhus and COVID-19 (and a CRP <10) – are likely to actually have typhoid, so the impact on the study’s power to assess efficacy endpoints in the subset with proven typhoid infection (culture positive) is unclear. Additionally, the sample size calculations assume treatment failure of 15%, but it is not clear whether this assumption refers to an estimated failure rate of treatment for typhoid infection specifically, or overall treatment failure in the context of an ITT analysis. Clarification here is needed. Finally, a 10% loss to follow-up out to 28 days is perhaps optimistic and should be justified.

**Minor comments**

 On page 6, under Secondary Objectives, the following is stated twice ‘To compare the time from onset of symptoms to treatment failure in patients in each treatment arm”

 A secondary endpoint (#5, page 6) is stated as “Positive culture of faeces sample for S. Typhi or S. Paratyphi” – suggest that the relevant timepoint(s) be explicitly stated

 Will enrolled patients be supplied with a thermometer so they can check their temperature at home?

 If a patient has a positive blood culture with antibiotic sensitivities that demonstrate resistance to the study medication(s), will a change to therapy before day 7 be instituted, or will this depend on clinical response?

 What is the reason for limiting the upper age to 65 years?

 An exclusion criterion is cardiac disease – does this extend to all types of cardiac disease, or is it only QT interval or arrhythmias?

 Page 8 states that children with adequate understanding may be able to sign the assent form, yet asper Page 10, this includes consent for DNA extraction for investigation of human genetic variables. I question the ethics of allowing children to consent to this aspect of the protocol.

 On page 9, under Follow-up trial visits, it is stated “For patients who had positive blood or stool culture for S. Typhi or S. Paratyphi at the time of admission…..” – since patients must have uncomplicated infection, I presume they won’t (usually) be admitted, so should this be “presentation” rather than “admission”?

 Bottom of 11/top P12 ‘Clinical data will be entered directly in tablets will be utilized from the various sites to enter data directly to the central database collection site at the Nepal CTU.” – something is missing from this sentence, which currently does not make sense.

 Further to my main comment above, the Discussion (page 14) also seems to mix potential impacts on suspected and proven typhoid infections.

 Will some children in the recruitment catchment potentially be vaccinated, and if so will this be documented?

Is the study design appropriate for the research question?

Partly

Is the rationale for, and objectives of, the study clearly described?

Yes

Are sufficient details of the methods provided to allow replication by others?

Yes

Are the datasets clearly presented in a useable and accessible format?

Not applicable

Reviewer Expertise:

Infectious diseases, study design

I confirm that I have read this submission and believe that I have an appropriate level of expertise to confirm that it is of an acceptable scientific standard, however I have significant reservations, as outlined above.

# Response

**Published:** 2021-11-08

**Authors:** Abhishek Giri

**Affiliations:** Oucru-Nepal, Nepal

We would like to very much thank Karin Leder for her comprehensive input that has definitely enhanced the presentation of the protocol in its clarity and focus.

 Below is our point-by-point response and suitable changes have also been made in the track-changed manuscript. Reviewer comments are in italics.

***Comments:***

*The rationale for performing this study to assess the efficacy of monotherapy versus combined therapy for the treatment of typhoid fever is well-justified, and the protocol is generally clear and sufficiently detailed.*

*However, I have suggested that the study design is only partly appropriate for the research question because patients with suspected typhoid fever will be included, thereby mixing outcomes of patients with typhoid and those with other conditions. I note that the main analysis will be as ITT, but that primary and secondary endpoints will be analyzed both as ITT and among the culture-confirmed population. I realize that the ITT design has practical and logistic advantages, but some non-typhoidal bacterial infections will also respond to these antibiotics. Consequently, since the main analysis is ITT, there is an apparent mismatch with the stated key primary outcome – to determine whether combination therapy is superior to azithromycin minor in preventing treatment failure in patients with uncomplicated typhoid fever. In fact, the ITT analysis will answer whether combination therapy is superior to azithromycin minor in preventing treatment failure in patients with suspected typhoid fever, but not specifically in those with uncomplicated typhoid fever. Both questions have clinical importance, but the precise main research question being addressed should be accurately stated and directly matched to the inclusion criteria and the primary endpoints (currently a mix of clinical and typhoid-specific microbiological criteria).*

*My concern about the precise research question also relate to the power calculations, where there is a lack of clarity regarding whether it is suspected or proven infection that serves as the main endpoint. No data are provided on the proportion of suspected cases that – after exclusion of malaria, dengue, scrub typhus and COVID-19 (and a CRP <10) – are likely to actually have typhoid, so the impact on the study’s power to assess efficacy endpoints in the subset with proven typhoid infection (culture positive) is unclear. Additionally, the sample size calculations assume treatment failure of 15%, but it is not clear whether this assumption refers to an estimated failure rate of treatment for typhoid infection specifically, or overall treatment failure in the context of an ITT analysis. Clarification here is needed. Finally, a 10% loss to follow-up out to 28 days is perhaps optimistic and should be justified.*

**Response:**

 We thank the reviewer for raising this very important issue and pointing out where there is a lack of clarity. In this RCT we are comparing the response of azithromycin alone and azithromycin plus cefixime in clinically suspected and confirmed typhoid fever. This is outlined in the title, but we have not outlined this clearly in the text (Page 5, Protocol, primary objective) and this now has been corrected in the text. We plan a sub-analysis of the culture-positive and culture-negative participants, but the primary analysis is the intention to treat. This represents the real group of patients that clinicians will be treating.

 Establishing the best endpoint in a typhoid treatment trial is not easy because of the challenges of case detection. Blood culture is the most widely accepted method of confirming the diagnosis but lacks sensitivity. In a systematic review, the sensitivity of blood culture in 529 true positive cases was 61% (95% CI 52-70%) compared with a bone marrow culture sensitivity of 96% (95% CI 92-99%) . ^1^ Although more sensitive than blood culture, bone marrow culture is not feasible for out-patient managed typhoid. Other diagnostic approaches using serology or blood PCR also lack sensitivity and specificity. ^2,3^ Many of the previous randomized controlled trials in typhoid fever have been criticized for restricting the analysis to blood culture-confirmed cases and not analyzing the outcome by intention to treat . ^4,5^

 Designing entry criteria to the study which therefore capture patients with typhoid fever is difficult. We know that there is an overlap in the clinical features of typhoid fever with other local infections. The clinical entry criteria in this RCT have been designed to try and optimize the proportion of patients who have typhoid fever. These criteria include clinical features consistent with an undifferentiated fever and the use of RDTs to exclude other common infections that may mimic typhoid. But it is evident that the patients recruited will include patients with blood culture-positive typhoid fever, patients with typhoid who are blood culture-negative, and patients with another infection whose clinical features are similar to typhoid.

 In a recently published RCT, the NUFIT study, comparing azithromycin and trimethoprim-sulfamethoxazole in undifferentiated febrile illness in Nepal, the entry criteria are close to this study. ^4^ In this study the proportion of patients with culture-positive typhoid fever was 87 (27%) of 326 participants. We do not know how many of the other participants had blood culture-negative typhoid fever. If this proportion of 27% is replicated in the ACT-SA study there will be approximately 200 culture-positive participants in each arm of the study.

 The estimated proportion of 15% of treatment failures with azithromycin was based on a review of the failure rates in the seven RCTs that were included in azithromycin systematic reviews. ^5^ The outcome in all of these studies was based on blood culture positive typhoid patients and the studies were not blinded. ^7-13^ Failures in the azithromycin arm varied between 0% and 18% across the different trials (Figure 1). Each of these trials used slightly different azithromycin regimens and varied in the outcome measure used. The only RCT to use the 7-day 20mg/kg/day regimen that we propose to use in this trial (and the largest RCT) ^13^ recorded a failure in 9.1% (13/142) of subjects. The next largest RCT which used an outcome definition close to the one which will be used in this study and an azithromycin dose of 10mg/kg/day for 7 days recorded a failure in 18% (11/62) of subjects. ^12^ The most recent of these trials was completed in Vietnam when there was no azithromycin resistance reported. ^13^

**Figure 1: **Random and fixed-effects meta-analysis of failure proportions with azithromycin monotherapy treatment of typhoid observed in earlier randomised controlled trials. ^7-13^ 

 Footnote: For individual studies, Pearson-Clopper confidence intervals are given. The combination was via the inverse variance method based on the logit-transform with a continuity correction of 0.5 in studies with zero cell frequencies. DerSimonian-Laird estimator for tau^2. 

 Sporadic reports of azithromycin are beginning to appear and in the review of sensitivity data across the four sites involved in this trial resistance to azithromycin was present in 48 (2%) of 3617 isolates. Based on this evidence we considered it reasonable to estimate a failure rate of 15% in the azithromycin arm.

 If it is the case that we have 200 blood culture-positive patients in each arm of the trial, this will give an 80% power to detect a difference of 15% failure in the azithromycin arm and 6% in the azithromycin and cefixime arm at the two-sided significance level of 5%. Notably, in the NUFIT study which was published after our analysis of failure rates in RCTs was compiled and which was conducted double-blind, the treatment failure proportion with azithromycin at 28 days was 23 (14%) of 163 in the intention to treat analysis and 11 (30%) of 37 among the culture-positive participants.

 In our experience in past typhoid treatment RCTs, the dropout rate has varied between 10 and 20%. This is why we chose a 10% dropout proportion in the ACT-SA study although we will need to monitor this closely.

  

1. Mogasale V,  Ramani E, Mogasale VV, Park JY. What proportion of *Salmonella *Typhi cases are detected by blood culture? A systematic literature review. Ann Clin Microbiol Antimicrob 2016; 15:32
2. Wijedoru L, Mallett S, Parry CM. Rapid diagnostic tests for typhoid and paratyphoid (enteric) fever (Review). Cochrane Database of Systematic Reviews 2017, Issue 5. Art CD008892
3. Moore CE, Pan-Ngum W, Wijedoru LPM, et al. Evaluation of the diagnostic accuracy of a typhoid IgM flow assay for the diagnosis of typhoid fever in Cambodian children usiang a Bayesian latent class model assuming an imperfect gold standard. Am J Trop Med Hyg 2014; 90:114-120.
4. Giri A, Karkey A, Dongol S, eta l. Trimethoprim-Suphamethoxazole versus azithromycin for the treatment of undifferentiated febrile illness in Nepal: A double-blind, randomized, placebo-controlled trial. Clin Infect Dis 2020; 73:e1478.
5. Effa EE, Burkiwa H. Azithromycin for treating uncomplicated typhoid and paratyphoid fever (enteric fever) (Review) Cochrane Database of Systematic Reviews 2008, Issue 4. Art CD006083
6. Effa EE, Lassi ZS, Critchley ZA, Garner P, Sinclair D, Olloaro P, Bhutta ZA. Fluoroquinolones for treating typhoid and paratyphoid fever (enteric fever) (Review) Cochrane Database of Systematic Reviews 2011, Issue 10. Art CD004530
7. Butler T, Sridhar CB, Daga MK, et al. Treatment of typhoid fever with azithromycin versus chloramphenicol in a randomized multicentre trial in India. *J Antimicrob Chemother* 1999; 44(2): 243-50.
8. Girgis NI, Butler T, Frenck RW, et al. Azithromycin versus ciprofloxacin for treatment of uncomplicated typhoid fever in a randomized trial in Egypt that included patients with multidrug resistance. *Antimicrob Agents Chemother* 1999; 43(6): 1441-4.
9. Chinh NT, Parry CM, Ly NT, et al. A Randomized Controlled Comparison of Azithromycin and Ofloxacin for Treatment of Multidrug-Resistant or Nalidixic Acid-Resistant Enteric Fever. *Antimicrob Agents Chemother *2000; 44(7): 1855-9.
10. Frenck RW, Jr., Nakhla I, Sultan Y, et al. Azithromycin versus ceftriaxone for the treatment of uncomplicated typhoid fever in children. *Clin Infect Dis* 2000; 31(5): 1134-8.
11. Frenck RW, Jr., Mansour A, Nakhla I, et al. Short-course azithromycin for the treatment of uncomplicated typhoid fever in children and adolescents. *Clin Infect Dis* 2004; 38(7): 951-7.
12. Parry CM, Ho VA, Phuong LT, et al. Randomized controlled comparison of ofloxacin, azithromycin, and an ofloxacin-azithromycin combination for treatment of multidrug-resistant and nalidixic acid-resistant typhoid fever. *Antimicrob Agents Chemother* 2007; 51:819-25
13. Dolecek C, Tran TP, Nguyen NR  et al. A multicenter randomised controlled trial of gatifloxacin versus azithromycin for the treatment of uncomplicated typhoid fever in children and adults in Vietnam. PLoS ONE 2008; 3:e2188.

***Minor comments***

*On page 6, under Secondary Objectives, the following is stated twice ‘To compare the time from onset of symptoms to treatment failure in patients in each treatment arm”*

- Thank you very much. We have now deleted the redundant portion.

*A secondary endpoint (#5, page 6) is stated as “Positive culture of feces sample for S. Typhi or S. Paratyphi” – suggest that the relevant timepoint(s) be explicitly stated*

- We have now added this point about explicitly stating the timepoint.

*Will enrolled patients be supplied with a thermometer so they can check their temperature at home?*

- Yes.

*If a patient has a positive blood culture with antibiotic sensitivities that demonstrate resistance to the study medication(s), will a change to therapy before day 7 be instituted, or will this depend on clinical response?*

- This will depend on the clinical response.

*What is the reason for limiting the upper age to 65 years?*

- With azithromycin administration, one of our earlier reviewers suggested this to play it safe to avoid QT prolongation problems in the elderly population.

*An exclusion criterion is a cardiac disease – does this extend to all types of cardiac disease, or is it only QT interval or arrhythmias?*

- All types including coronary artery disease.

*Page 8 states that children with adequate understanding may be able to sign the assent form, yet as per Page 10, this includes consent for DNA extraction for investigation of human genetic variables. I question the ethics of allowing children to consent to this aspect of the protocol.*

- We agree. And as stated in the protocol we will rigorously follow the local ethical guidelines.

*On page 9, under Follow-up trial visits, it is stated “For patients who had positive blood or stool culture for S. Typhi or S. Paratyphi at the time of admission…..” – since patients must have an uncomplicated infection, I presume they won’t (usually) be admitted, so should this be “presentation” rather than “admission”?*

- Thank you very much for picking this up. We have now changed it to presentation.

*Bottom of 11/top P12 ‘Clinical data will be entered directly in tablets will be utilized from the various sites to enter data directly to the central database collection site at the Nepal CTU.” – something is missing from this sentence, which currently does not make sense.*

- Thank you very much. We have now tried to clarify this obvious confusion which the Reviewer kindly picked up.

*Further to my main comment above, the Discussion (page 14) also seems to mix potential impacts on suspected and proven typhoid infections.*

- We have now tried to make this more clear in the Discussion including as addressed in the main comment section of this Reviewer.

*Will some children in the recruitment catchment potentially be vaccinated, and if so will this be documented?*

- Yes.
